# Supplementary material for: Drosophila USP22/nonstop polarizes the actin cytoskeleton during collective border cell migration
Source: J Cell Biol. 2021 May 14;220(7):e202007005. doi: 10.1083/jcb.202007005 (PMC8129793; doi:10.1083/jcb.202007005)
Supplement: Table S1 — summarizes data showing the association of Not and Ada2b with the promoters of Hippo pathway genes. [file JCB_202007005_TableS1.docx]

|  | Number of times promoter binding was identified by ChIP-Seq in biological replicates (Lin et al 2014) | |
| --- | --- | --- |
|  | **ChIP: Ada2b** | **ChIP: Not** |
| Hippo pathway gene |  |  |
| *hpo* | 4 out of 4 | 2 out of 2 |
| *crb* | 3 out of 4 | 2 out of 2 |
| *kibra* | 2 out of 4 | 2 out of 2 |
| *zyxin* | 2 out of 4 | 2 out of 2 |
| *warts* | 0 out of 4 | 0 out of 2 |
| *mer* | 0 out of 4 | 1 out of 2 |
| *ex* | 0 out of 4 | 2 out of 2 |
|  |  |  |
